# Fate of *Listeria monocytogenes* in the Presence of Resident Cheese Microbiota on Common Packaging Materials

**DOI:** 10.3389/fmicb.2020.00830

**Published:** 2020-05-15

**Authors:** Pierluigi Di Ciccio, Selene Rubiola, Maria Ausilia Grassi, Tiziana Civera, Francesco Abbate, Francesco Chiesa

**Affiliations:** ^1^Department of Veterinary Science, University of Turin, Turin, Italy; ^2^Department of Veterinary Sciences, University of Messina, Polo Universitario della Annunziata, Messina, Italy

**Keywords:** *Listeria monocytogenes*, cross-contamination, packaging materials, dairy products, cold storage, food handling

## Abstract

Literature data regarding the survival of microorganisms on materials used for food package purposes are scarce. The aim of the current study is to assess the survival of *Listeria monocytogenes* on different packaging materials for dairy products during extended storage at different temperatures. Three packaging materials (5 × 5 cm) were contaminated with a cocktail of five strains of *Listeria monocytogenes* suspended in a cheese homogenate, including the cheese’s native microbial population. Contaminated samples were incubated at 37°, 12°, and 4°C and periodically analyzed up to 56 days. The evolution of the total viable count and pathogen population was evaluated. At 37°C, the results showed that *Listeria monocytogenes* was no longer detected on polyethylene-coated nylon (B) by day 4 and on polyethylene-coated parchment (A) and greaseproof paper (C) by day 7. Interestingly, the initial cell population (ranging between 2.5 and 2.7 log CFU/cm^2^) of *Listeria monocytogenes* increased to 3 log CFU/cm^2^ within 4 days of storage at 12°C on A and C. During storage, the number remained fairly constant at 12°C and 4°C on two materials (A–C) and decreased slowly on the third one (B). This study shows that survival of *Listeria monocytogenes* on packaging materials for dairy products will be higher when stored at 4 or 12°C compared to 37°C. The survival of *Listeria monocytogenes* on the packaging materials raises concerns of cross-contamination during food handling and preparation at catering and retail premises and within the home, highlighting the importance of treating the packaging materials as a potential source of cross-contamination. These initial findings may aid in quantifying risks associated with contamination of food packaging materials.

## Introduction

*Listeria monocytogenes* (*L. monocytogenes*) is of interest because infection is often associated with a high mortality rate, particularly among the elderly (Information for Health Professionals and Laboratories | Listeria | CDC, 2018). Based on the European Union summary report on trends and sources of zoonoses, zoonotic agents, and food-borne outbreaks in 2017, there has been a statistically significant increased trend of listeriosis in the European Union (EU). Specifically, the numbers of confirmed human cases of listeriosis were 2,480 in 2017.

The EU notification rate was 0.48 cases per 100,000 populations, whereas the EU case fatality was 13.8%. Interestingly, most listeriosis cases have been domestically acquired (The European Union summary report on trends and sources of zoonoses, zoonotic agents, and food−borne outbreaks in 2017–2018 – EFSA, 2018 Journal – Wiley Online Library).

The first human case of listeriosis associated with the consumption of cheese was reported by Linnan et al. (1988). Since then, several outbreaks caused by the consumption of cheese have occurred worldwide (Rios and Dalgaard, 2018). *L. monocytogenes* represent a significant issue for the food industry and health service management (Le et al., 2014). *L. monocytogenes* was found most frequently in soft and semi-soft cheese (Schoder et al., 2014; Morandi et al., 2019).

The pathogen can survive in harsh conditions found in the food processing industry, and it can grow at refrigeration temperatures (Havelaar et al., 2010; Carpentier and Cerf, 2011). Additionally, *L. monocytogenes* form biofilms on common surfaces found in the food processing environment (Wilks et al., 2006; Bonaventura et al., 2008; Martinon et al., 2012). Cross-contamination of foods with *L. monocytogenes* following contact with inert surfaces has been reported (de Candia et al., 2015; Erickson et al., 2015).

Data regarding the contamination and microbial populations on package materials are scarce in the literature. Siroli et al. (2017), evaluated the effect of packaging materials for fruits and vegetables on the survival of different microbial species during storage. The authors demonstrated that cardboard materials, if stored in an environment with low relative humidity, reduce the potential cross-contamination of food due to a quicker viability loss by microorganisms compared to plastic materials. As for as the packaging materials are concerned, the EU Regulation EC n° 852/2004 (Eur-Lex, 2014) in the Annex II, Chapter X, established that “all materials used for wrapping and packaging must not be a source of contamination.” It has been known that most foodborne disease episodes occur as a result of errors during food preparation at home (The European Union summary report on trends and sources of zoonoses, zoonotic agents, and food−borne outbreaks in 2017–2018 – EFSA, 2018 Journal – Wiley Online Library). Handling of the contaminated packaging materials may lead to cross-contamination of environments (work surfaces, refrigerator surfaces, etc.), and they may represent a source of contamination during food preparation at retail premises and within the home.

In regard to this, the current study aimed to (i) evaluate the survival of *L. monocytegenes* in the presence of cheese resident microbiota on packaging materials commonly used for dairy products and (ii) compare differences of *L. monocytogenes* behavior when exposed to different storage temperatures.

## Materials and Methods

### Microbial Strains and Inoculum Preparation

*L. monocytogenes* was used in this study as the model microbial pathogen for dairy products. All *L. monocytogenes* strains were obtained from the Bacterial Culture Collection at the Department of Veterinary Science, University of Turin. The *L. monocytogenes* inoculum used in this study was comprised of a mixture of five strains isolated from dairy products (Table 1). In particular, the strains used in this study consisted of two *L. monocytogenes* isolates belonging to sequence type (ST) 6 and two and one strain of *L. monocytogenes* belonging to ST 325 and ST 38, respectively. These STs were chosen, as they are more often found to persist in local (Piedmont) dairy industries (Lomonaco et al., 2018). *In silico* MLST analysis was performed using tools in the BIGSdb-Lm database.^1^ A single colony from each strain was activated twice in 10 mL of Brain Heart Infusion (BHI, Oxoid, Milan) by incubation at 37°C for 24 h. Cell suspensions were individually centrifuged (4,600 × g, 15 min, 4°C), and the supernatant was discarded. Cultures were then washed three times with phosphate buffered saline (PBS – pH 7.3, Sigma−Aldrich S.r.l., Milan, Italy) and diluted with PBS resulting in a final concentration of ∼10^8^ CFU/mL by reading the optical density (OD) level at 550 nM (Uvikon-930 spectrophotometer – Kontron instrument, Germany). The final washed pellets of individual strains were re-suspended in 10 mL of pasteurized ultra-filtered milk (pH: 6.6–6.7) and stored for 72 h at 4°C in order to habituate cell cultures to an environment typical of milk products stored at cool temperatures (Angelidis et al., 2002). Ten milliliters of each strain were mixed to obtain a five-strain cocktail which was then serially diluted in a non-sterile cheese homogenate in order to reach a final target level ranging between 2.5–2.7 log CFU/cm^2^.

**TABLE 1:** *L. monocytogenes* strains used in this experiment.

Year	GenBank assembly accession	ST	Clonal complex	Isolation source	Sero- group
2008	GCA_002523555.1	ST38	CC101	Dairy environment	Iia
2009	GCA_002523545.1	ST325	CC31	Dairy environment	Iia
2009	GCA_002523515.1	ST6	CC6	Dairy environment	IVb
2009	GCA_002523505.1	ST6	CC6	Dairy environment	IVb
2009	GCA_002523605.1	ST325	CC31	Dairy environment	IIa

### Cheese Homogenate

*Toma-Piemontese*, an artisanal protected denomination origin (PDO) cheese (pH 6.0–7.0), was used in this experiment. This PDO cheese is produced in Piedmont (Northwest Italy) from raw milk with the addition of selected starter cultures. The ripening process is carried out under controlled conditions of temperature (6–10°C) and relative humidity (85%) for ∼60 days (Fortina et al., 2003). The production and ripening process depends on the natural microbial population present in the raw milk and starter culture. The bacterial composition of the commercial starter mixes generally contains thermophilic and mesophilic lactic bacteria, such as *Enterococcus faecium*, *Lactobacillus plantarum*, *Streptococcus thermophilus*, *Lactobacillus delbrueckii* spp. *bulgaricus* and spp. *lactis*, *Lactobacillus helveticus*, *Lactococcus lactis* spp. *cremoris*, *Lactococcus lactis* spp. *lactis*, and *Leuconostoc* spp.

In particular, a cheese homogenate was made by using 10% of *Toma-Piemontese* and 90% of sterile PBS, and the resulting sample was homogenized for 1 min using a stomacher (Seward Ltd., London, United Kingdom). Before use, the initial bacteria of this cheese were enumerated and identified. In detail, 10 g of cheese was blended with 90 mL buffered peptone water (BPW – Oxoid, Basingstoke, United Kingdom) and was serially diluted in sterile PBS. Finally, an aliquot of 100 μL was placed in duplicate on different agars: plate count agar and incubated at 30°C (48–72 h) for determination of the total bacterial count (TVC); bile esculin azide agar (Biolife, Milan) and incubated at 37°C (24–48 h) for enterococci; M17 agar (Oxoid, Milan) and incubated at 25°C and 44°C (48 h) for mesophilic and thermophilic cocci, respectively; MRS agar (Oxoid, Milan) at pH 5.8 and incubated at 30°C and 42°C (48–72 h) for mesophilic and thermophilic lactobacilli, respectively. In addition, the cheese was checked for the presence of *L. monocytogenes* according to the ISO 11290-1 2017, with minor modification. In detail, 25 g of *Toma-Piemontese* were added to half-Fraser broth (225 mL – Merck, Germany). It was homogenized in a stomacher and incubated at 30 ± 1°C (24 h). After incubation, 0.1 mL of the half-Fraser broth was inoculated in Fraser broth (10 mL) and incubated at 37°C (48 ± 2 h). After incubation, 1 mL of culture was spread onto both the chromogenic listeria agar base (Ocla – Oxoid, Basingstoke, United Kingdom) and the agar listeria ottaviani agosti base (Aloa – Merck, Germany) and incubated at 37°C (24–48 h). Absence of typical *L. monocytogenes* colonies on these selective agars (Angelidis et al., 2015) confirmed that the cheese homogenate was ready to inoculate with the five-strain *L. monocytogenes* cocktail.

### Inoculation of Packaging Materials and Scanning Electron Microscopy (SEM) Analyses

Three materials with different physico-chemical properties were provided by INALPI S.p.A (Moretta, Cuneo – Italy). Polyethylene-coated parchment (designated as A), polyethylene-coated nylon (designated as B), and greaseproof paper (designated as C) were used in this study. These materials are widely used in food packaging for dairy products.

All package materials were cut into squares (5 by 5 cm) and exposed to a UV lamp at 254 nM (30 min) for disinfection before inoculation (Bachmann et al., 1976). Subsequently, 0.5 mL of previously described *L. monocytogenes* culture cocktail (4 log CFU/mL) in non-sterile cheese homogenate was applied to the surface of each packaging piece (5 × 5 cm) in order to reach a final target level ranging between 2.5 and 2.7 log CFU/cm^2^. The inoculated surfaces were air-dried for 30 min at room temperature (22 ± 2°C), and they were then stored aerobically at 37°C (with relative humidity up to 80%), 12°C (with relative humidity up to 60%), and 4°C (with relative humidity up to 60%) for 56 days. Non-inoculated control samples (cheese homogenate without *Listeria monocytogenes* culture cocktail) were kept and analyzed at the end of experiment. Furthermore, analyses of packaging surfaces by scanning electron microscope (SEM) technique were performed in order to obtain information on their microstructure.

### Microbiological Analysis

In order to quantify the microbial population persisting on each inoculated packaging material sample, a random selection of three samples of each material was removed from their respective incubators immediately prior to testing (T0). The bacteria on the inoculated surfaces were enumerated after 24 h (T1), 48 h (T2), 4 days (T4), and weekly until the 35 days of storage for *L. monocytogenes* and TVC. The last time analysis was carried out 3 weeks later (56 days of storage). Regarding the sample stored at 37°C, no evidence of *L. monocytogenes* was found after 7 days, therefore testing on this sample was suspended at that point. For enumeration of *L. monocytogenes* and mesophilic aerobic bacteria, the samples were transferred into sterile stomacher bags (Lab Plas Inc., Sainte-Julie, QC, Canada) containing 10 mL of PBS (pH 7.3 – Oxoid, Basingstoke, United Kingdom) and homogenized for 2 min with a stomacher (Seward Ltd., London, United Kingdom). Appropriate dilutions were then surface-plated onto both Ocla (Oxoid, Basingstoke, United Kingdom) and tryptone soy agar (TSA – Oxoid, Basingstoke, United Kingdom). The Ocla plates were incubated at 37°C for 48 h, whereas TSA plates were incubated at 30°C for 24/48 h. After incubation, colonies were manually counted. The *L. monocytogenes* and TVC counts for each sample were expressed as Log_10_ colony forming unit (CFU)/cm^2^. The enumeration limit of the analysis was 0.4 CFU/cm^2^. Sampling was concluded for materials that had counts below the enumeration limit for at least two consecutive sampling dates. Finally, to confirm the absence *L. monocytogenes* cells, a qualitative method of *L. monocytogenes* detection according to the ISO 11290-1, 2017 was followed on these samples. The experiments were performed three times independently, and the data were statistically analyzed.

### Statistical Analysis

Average log-transformed values were compared with Kruskal-Wallis tests followed by Dunn’s post-test, for more than two groups of data. Analyses were performed using XLSTAT (Addinsoft) and GraphPad Prism 7.0 (GraphPad Software). A *p*-value < 0.05 was considered significant.

## Results

### Enumeration of Bacteria of *Toma Piemontese*

The TVC showed mean counts of 6.71 ± 0.12 Log CFU/g. The mean of enterococci count showed 4.78 ± 0.10 Log CFU/g. Counts revealed 5.45 ± 0.21 and 3.94 ± 0.14 for cocci mesophilic and thermophilic, respectively; whereas the mean value for lactobacilli counts (mesophilic and thermophilic) were 5.57 ± 0.10 and 4.58 ± 0.12, respectively.

### Survival of *L. monocytogenes* During Storage of Inoculated Packaging Materials at 37°, 12°, and 4°C

**FIGURE 1:**
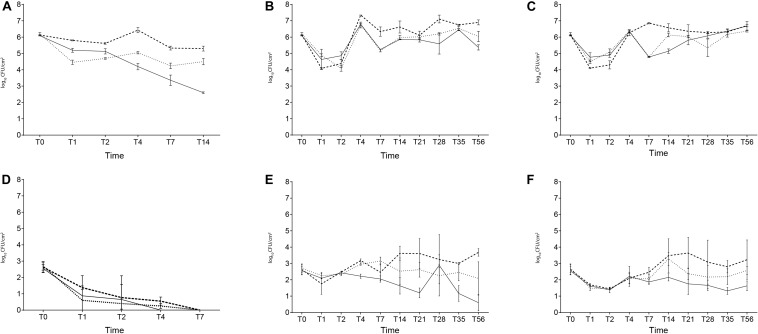
The microbial populations of TVC **(A–C)** and *L. monocytogenes* **(D–F)** recorded during storage at 37°C **(A,D)**, 12°C **(B,E)**, and 4°C **(C,F)**. Dotted line, polyethylene-coated parchment; solid line, polyethylene-coated nylon; dashed line, greaseproof paper.

The behavior of *L. monocytogenes* on the packaging materials stored at 37°C is shown in Figure 1D. At 37°C, the populations of *L. monocytogenes* decreased to levels below the enumeration limit (0.4 CFU/cm^2^) after 4 days of storage in B and 7 days of storage in A and C.

Concerning TVC, the initial inoculum level (6.2 log CFU/cm^2^) decreased from 5.3 (C) to 2.6 log CFU/cm^2^ (B). In short, TVC aligned closely with the trend of the pathogen up to T7 (Figure 1A). The behavior of *L. monocytogenes* on the packaging materials stored at 12°C is shown in Figure 1E.

At 12°C, *L. monocytogenes* numbers remained high enough to be enumerated using direct plating throughout the entire length of the study (56 days) on all packaging materials. *L. monocytogenes*, in fact, was recovered from all the tested materials, with average counts ranging from 0.4 log CFU/cm^2^ (B) to 3.6 log CFU/cm^2^ (C), respectively.

*L. monocytogenes* population decreased independently of the packaging materials after 24 h (T1). However, a slight increase in *L. monocytogenes* cell density was observed after 48 h (T2). Specifically, initial levels of *L. monocytogenes* increased to 3 log CFU/cm^2^ within 4 days of storage (T4) at 12°C on A and C.

Cell density on C after 56 days at 12°C was 1 log CFU/cm^2^ higher than initial levels (2.6 log CFU/cm^2^), whereas pathogen populations on A and B were 0.6 to 1.9 log CFU/cm^2^ lower than initial levels. Regarding TVC, after 56 days (T56) the initial inoculum level increased to 6.9 log CFU/cm^2^ in C and decreased on A and B, ranging from 6 (A) to 5.3 (B) log CFU/cm^2^, respectively (Figure 1B).

The behavior of *L. monocytogenes* on the packaging materials stored at 4°C is shown in Figure 1F.

At 4°C, pathogen was enumerated until the end of study (56 days) on all packaging materials, with average counts ranging from 1.6 log CFU/cm^2^ (B) to 3.2 (C) log CFU/cm^2^, respectively.

At 4°C, the *L. monocytogenes* populations decreased independently on the packaging materials after 24 h (T1) and 48 h (T2). Survivors of the pathogen after 56 days showed a count of 3.2 log CFU/cm^2^, 2.5 log CFU/cm^2^ and 1.6 log CFU/cm^2^ in C, A, and B, respectively.

Cell density on C after 56 days at 4°C was 0.6 log CFU/cm^2^ higher than initial levels (2.6 log CFU/cm^2^), whereas pathogen populations on A and C were 0.1–0.9 log CFU/cm^2^ lower than initial levels.

As far as TVC is concerned, the initial inoculum level of 6.2 log CFU/cm^2^ increased on all materials, ranging from 6.7 (C) to 6.4 log CFU/cm^2^ (A) (Figure 1C).

Overall, regardless of storage temperature, packaging materials most and least conducive to *L. monocytogenes* survival in cheese residues were C and B, respectively.

The absence of typical *L. monocytogenes* colonies on non-inoculated control samples (cheese homogenate without *L. monocytogenes* culture cocktail) was confirmed according to ISO 11290-1 (2017) at the end of experiment (56 days) on all packaging materials.

### Statistical Analysis

The average counts of *L. monocytogenes* at all sampling points, 12 and 4°C, were analyzed: in particular, highly significantly different *p*-values (<0.0001) were observed between B and C irrespective of the temperature; significantly different *p*-values (<0.05) were shown between A and B as well as A and C, irrespective of the temperature, and between A and C at 12°C.

### SEM Analysis

**FIGURE 2:**
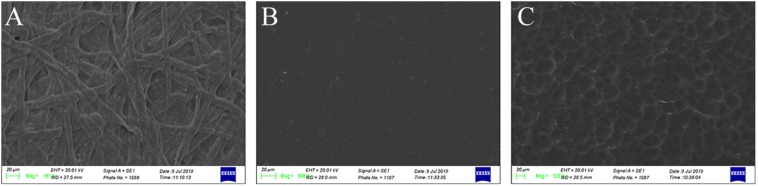
SEM images of polyethylene-coated parchment (designated as **A**), polyethylene-coated nylon (designated as **B**), and greaseproof paper (designated as **C**). In **(A,C)**, package materials the presence of fibers and micro-pores is evident. By contrast, B package material showed a smooth, plane, and homogeneous surface without holes or micro-pores able to entrap microorganisms.

The SEM analysis was carried out on the three packaging materials used in this study to detect the presence or absence of pores and holes that are able to entrap *L. monocytogenes* cells. In regard to this, the microstructure of the polyethylene-coated parchment (A) and greaseproof paper (C) showed fibers and pores, respectively. By contrast, polyethylene-coated nylon (B) showed a smooth and homogeneous surface without holes able to entrap microbial cells (Figure 2).

## Discussion

This study evaluated the survival of *L. monocytogenes* with resident cheese microbiota on common packaging materials for dairy products at different storage temperatures. Currently, there are no studies to our knowledge that have examined the effect of different storage conditions and packaging material type on *L. monocytogenes*. It is well known that the microbial population differs greatly between cheeses prepared from pasteurized and unpasteurized milk. Generally, the microbiota of cheeses contain mesophilic lactic acid bacteria (LAB), which grow optimally at temperatures between 30 and 37°C (Callon et al., 2011). The microbiota interacts strongly with *L. monocytogenes* generating a competitive environment that regulates its survival (Powell et al., 2004; Schirmer et al., 2013). In light of this, based on our results, the numbers of presumptive LAB increased over time, and the microbial interactions taking place in samples stored at 37°C may promote the antagonistic effect of the starter culture against *L. monocytogenes.* In contrast, the survival of *L. monocytogenes* was observed at cool temperatures until the end of experiment (56 days). It is no surprise that survival was observed at 4°C and 12°C for long period of time, as *L. monocytogenes* has been demonstrated to be persistent under less than ideal conditions (Tompkin, 2002; Autio et al., 2003; Vogel et al., 2010) and is able to grow at temperatures below 1°C (Junttila et al., 1988). Another study on the survival of *L. monocytogenes* in artificially contaminated cheese during storage at 4 and 12°C, the authors showed a higher survival at 4 and 12°C as opposed to during storage at a higher storage temperature (Angelidis et al., 2010).

In nature, the *L. monocytogenes* contamination of foods may involve more than one strain of the microorganism (Danielsson-Tham et al., 1993; Navas et al., 2007; Zilelidou and Skandamis, 2018). For this reason, a simple inoculation model composed of five strains from dairy products in non-sterile cheese homogenate to simulate the real conditions in the packaging of dairy products was used in this experiment. As for as *L. monocytogenes*, strains belonging to serotype 4b are common in clinical cases whereas strains belonging to serotype 1/2a are, commonly, isolated from food environment (Poimenidou et al., 2018). Additionally, dairy products have been linked to both invasive and non-invasive listeriosis and strains belonging to serotype 4b represent the majority of the outbreak isolates (Melo et al., 2015). Based on our results, survival of *L. monocytogenes* varied among packaging materials for dairy products as well as at different storage temperatures.

Siroli et al. (2017), evaluated the effect of the two packaging materials (cardboard and plastic materials) for fruits and vegetables on the survival of different spoilage and pathogenic microorganisms during the storage at environmental temperature (1, 8, 24, and 48 h). Briefly, in this study, a reduction of microbial population of the pathogens independently of the inoculation level and packaging type was observed (Siroli et al., 2017). In particular, the reduction of microbial populations (spoilage and pathogenic bacteria) was faster in cardboard than plastic materials. By contrast, molds were able to grow in the presence of high humidity (water) during the storage.

Our work is the first study to provide information on the survival of *L. monocytogenes* in cheese residues on the surface of common packaging materials for dairy products. We attempted to identify the packaging types of materials that can allow *L. monocytogenes* to survive during long-term storage (56 days) at different temperatures (4, 12, and 37°C). Additionally, this choice of temperature was informed by the desire to simulate storage in a setting typical of both a retail establishment and a household. In particular, we simulated a temperature abuse that may allow a microbial proliferation both on the packaging materials and the foods (Kusumaningrum et al., 2003; Ndraha et al., 2018). Previous studies have shown that many domestic refrigerators operate at temperatures above those recommended (Azevedo et al., 2005; Lagendijk et al., 2008; Vegara et al., 2014). Thus, the inoculated surfaces were stored at 4°C (recommended temperature) or 12°C (temperature typical of many household refrigerators). The optimum growth temperature for *L. monocytogenes* (37°C) was included in this investigation. In short, the results indicate that *L. monocytogenes* may survive on food packaging materials with cheese residues for long periods, especially at 12 and 4°C. *L. monocytogenes* contaminated foods or packages found in domestic refrigerators may directly contaminate other stored foods or attach to and persist on the interior surfaces of the refrigerators. The most probable explanation for the long-term survival of *L. monocytogenes* on various soiled packaging materials is the ability to form biofilms. As suggested by the literature regarding survival of *L. monocytogenes* in harmful environments, biofilms could be an important factor concerning the survival of the pathogen in adverse environments (Bridier et al., 2015). Biofilm is an accumulation of bacterial cells on a surface that leads to the formation of complex structures that aid in survival of bacteria (Di Ciccio et al., 2015). Additionally, cheese residues on wet surfaces can facilitate biofilm formation (Harvey et al., 2007).

In general, irrespective of storage temperature, packaging materials most and least conducive to *L.monocytogenes* survival in cheese residues were greaseproof paper (C) and polyethylene-coated nylon (B), respectively. Specifically, at 12 and 4°C, after 56 days, *L. monocytogenes* was recovered from all the tested materials, with different bacterial counts. This variability may be related to the microtopography of different packaging materials selected for this study. The different physical-chemical properties and microstructure of materials tested may affect the attachment of *L. monocytogenes* on B compared to A and C. The roughness and porousness of surfaces and environmental conditions can affect the survival of microorganisms (Chmielewski and Frank, 2003; Montibus et al., 2016). Packaging materials can come into contact with an array of microorganisms in the retail establishment or a household during storage and food preparation that result in the contamination with spoilage or pathogenic bacteria. In fact, the microbial contamination of the packaging materials has been reported by several authors (Burgess et al., 2005; Mafu et al., 2011; Ismaïl et al., 2013; Chiesa et al., 2014). We hypothesized that *L. monocytogenes* cells in porous materials, such as polythene/parchment paper (A) and parchment paper (C), may promote adhesion and microbial biofilm formation. However, further studies are needed to verify this ability of the three packaging materials used in this experiment in relation to different environmental conditions. Cross-contamination of *L. monocytogenes* from surfaces to food products has been described (Kusumaningrum et al., 2003; Lin et al., 2006; Wilks et al., 2006). In a study carried out by Patrignani et al. (2016), the role of the packaging material (such as cardboard and plastic) in the cross-contamination of packed peaches was evaluated. These authors showed that the use of cardboard, compared to plastic, can prevent the cross-contamination from packaging to fruit (Patrignani et al., 2016). Conversely, in our study, the plastic material (polyethylene-coated nylon designated as B) was the best material to reduce the potential of *L. monocytogenes* survival compared to the two paper-based packaging (polythene/parchment paper designated as A and parchment paper designated as C). As it pertains to ready-to-eat food products such as soft cheeses, the Regulation (EC) 2073/05 on microbiological criteria for foodstuffs established that RTE foods “must not exceed the limit of 100 cfu/g for *L. monocytogenes* at any point during their shelf life.” Food business operators, in fact, must have evidence for each product to show that this pathogen does not exceed this limit throughout the entire shelf life. *L. monocytogenes* on soiled food packaging could pose a risk of cross-contamination because it can come into contact with all types of items, such as gloves, workbenches, surfaces of refrigerators, and different utensils during food preparation. Regarding this specific concern, incidences of listeriosis can be managed by a proper education of retailers and consumers helping to improve the good practices during food handling. Based on the results of the current study, the important point is that the *L. monocytogenes* was able to survive for long periods of time in the presence of cheese residues placed on the three food packaging materials stored at cool temperatures (12 or 4°C) in contrast to a significantly shorter survival period observed at the optimum growth temperature (37°C) for *L. monocytogenes*. In addition, the selection of polyethylene-coated nylon (B) may be desirable as packaging material for dairy products to reduce the potential survival of the pathogen. The knowledge on the survival of *L. monocytogenes* in the presence of resident cheese microbiota on common packaging materials may provide useful information in quantifying risks associated with contamination of food packaging materials for dairy products.

## Conclusion

This research was carried out to evaluate the behavior of *L. monocytogenes* on common packaging materials for dairy products during storage at different temperatures. *L. monocytogenes* was chosen as the target bacteria due to its importance in food safety. Our study has shown that, *L. monocytogenes* was more rapidly inactivated at 37°C and bacterial counts were below the enumeration limit (0.4 CFU/cm^2^) on all packaging materials by day 7 of storage. On the contrary, all three packaging materials can harbor *L. monocytogenes* for long periods of time, if stored at cool temperatures. Briefly, survival of *L. monocytogenes* on packaging materials is influenced by storage conditions and package type. Further studies are necessary to evaluate the ability of *L. monocytogenes* to survive and persist as biofilm on a wider range of food package materials in relation to storage and distribution conditions. These initial findings may aid in quantifying risks associated with contamination of food packaging materials.

## Data Availability Statement

All datasets generated for this study are included in article/Supplementary Material.

## Author Contributions

PD designed the experiments and wrote the manuscript. SR carried out the experiments in the lab. MG assisted the sample analyses. TC supervised the experiments and approved the final manuscript. FA carried out the SEM analysis and wrote the manuscript. FC analyzed and interpreted the data and reviewed the final manuscript.

## Conflict of Interest

The authors declare that the research was conducted in the absence of any commercial or financial relationships that could be construed as a potential conflict of interest.

## Footnotes

1 http://bigsdb.pasteur.fr/listeria/listeria.html

## References

[B1] Angelidis A. S. Boutsiouki P. Papageorgiou D. K. (2010). Loss of viability of *Listeria monocytogenes* in contaminated processed cheese during storage at 4, 12 and 22°C. *Food Microbiol.* 27 809–818. 10.1016/j.fm.2010.04.017 20630324

[B2] Angelidis A. S. Kalamaki M. S. Georgiadou S. S. (2015). Identification of non-*Listeria* spp. bacterial isolates yielding a β-D-glucosidase-positive phenotype on agar listeria according to ottaviani and agosti (ALOA). *Int. J. Food Microbiol.* 193 114–129. 10.1016/j.ijfoodmicro.2014.10.022 25462931

[B3] Angelidis A. S. Smith L. T. Smith G. M. (2002). Elevated carnitine accumulation by *Listeria monocytogenes* impaired in glycine betaine transport is insufficient to restore wild-type cryotolerance in milk whey. *Int. J. Food Microbiol.* 75 1–9. 10.1016/s0168-1605(02)00005-3 11999105

[B4] Autio T. Keto-Timonen R. Lundén J. Björkroth J. Korkeala H. (2003). Characterisation of persistent and sporadic *Listeria monocytogenes* strains by pulsed-field gel electrophoresis (PFGE) and amplified fragment length polymorphism (AFLP). *Syst. Appl. Microbiol.* 26 539–545. 10.1078/072320203770865846 14666982

[B5] Azevedo I. Regalo M. Mena C. Almeida G. Carneiro L. Teixeira P. (2005). Incidence of *Listeria* spp. in domestic refrigerators in Portugal. *Food Control* 16 121–124. 10.1016/j.foodcont.2003.12.006

[B6] Bachmann R. Bearda P. Stoidl W. Brandli G. Rieder R. (1976). *Ultraviolet Radiation Source Including Temperature Control and Pressure Control Operating Means.* Available at: https://patents.google.com/patent/US3971968A/en (accessed February 3, 2020).

[B7] Bonaventura G. D. Piccolomini R. Paludi D. D’Orio V. Vergara A. Conter M. (2008). Influence of temperature on biofilm formation by *Listeria monocytogenes* on various food-contact surfaces: relationship with motility and cell surface hydrophobicity. *J. Appl. Microbiol.* 104 1552–1561. 10.1111/j.1365-2672.2007.03688.x 18194252

[B8] Bridier A. Sanchez-Vizuete P. Guilbaud M. Piard J.-C. Naïtali M. Briandet R. (2015). Biofilm-associated persistence of food-borne pathogens. *Food Microbiol.* 45 167–178. 10.1016/j.fm.2014.04.015 25500382

[B9] Burgess F. Little C. L. Allen G. Williamson K. Mitchell R. T. (2005). Prevalence of *Campylobacter*, *Salmonella*, and *Escherichia coli* on the external packaging of raw meat. *J. Food Prot.* 68 469–475. 10.4315/0362-028X-68.3.469 15771168

[B10] Callon C. Saubusse M. Didienne R. Buchin S. Montel M.-C. (2011). Simplification of a complex microbial antilisterial consortium to evaluate the contribution of its flora in uncooked pressed cheese. *Int. J. Food Microbiol.* 145 379–389. 10.1016/j.ijfoodmicro.2010.12.019 21255857

[B11] Carpentier B. Cerf O. (2011). Review — Persistence of *Listeria monocytogenes* in food industry equipment and premises. *Int. J. Food Microbiol.* 145 1–8. 10.1016/j.ijfoodmicro.2011.01.005 21276634

[B12] CDC (2018). *Information for Health Professionals and Laboratories | Listeria* |. Available at: https://www.cdc.gov/listeria/technical.html (accessed February 3, 2020).

[B13] Chiesa F. Lomonaco S. Nucera D. Garoglio D. Dalmasso A. Civera T. (2014). Distribution of *Pseudomonas* species in a dairy plant affected by occasional blue discoloration. *Ital. J. Food Saf.* 3:1722. 10.4081/ijfs.2014.1722 27800364 PMC5076691

[B14] Chmielewski R. A. N. Frank J. F. (2003). Biofilm formation and control in food processing facilities. *Compr. Rev. Food Sci. Food Saf.* 2 22–32. 10.1111/j.1541-4337.2003.tb00012.x 33451238

[B15] Danielsson-Tham M.-L. Bille J. Brosch R. Buchrieser C. Persson K. Rocourt J. (1993). Characterization of *Listeria* strains isolated from soft cheese. *Int. J. Food Microbiol.* 18 161–166. 10.1016/0168-1605(93)90220-B 8494682

[B16] de Candia S. Morea M. Baruzzi F. (2015). Eradication of high viable loads of *Listeria monocytogenes* contaminating food-contact surfaces. *Front. Microbiol.* 6:733 10.3389/fmicb.2015.00733 PMC4503923 26236306

[B17] Di Ciccio P. Vergara A. Festino A. R. Paludi D. Zanardi E. Ghidini S. (2015). Biofilm formation by *Staphylococcus aureus* on food contact surfaces: relationship with temperature and cell surface hydrophobicity. *Food Control* 50 930–936. 10.1016/j.foodcont.2014.10.048

[B18] EFSA (2018). The European Union summary report on trends and sources of zoonoses, zoonotic agents and food-borne outbreaks in 2017. *EFSA J.* 6:5500 10.2903/j.efsa.2018.5500 PMC7009540 32625785

[B19] Erickson M. C. Liao J. Cannon J. L. Ortega Y. R. (2015). Contamination of knives and graters by bacterial foodborne pathogens during slicing and grating of produce. *Food Microbiol.* 52 138–145. 10.1016/j.fm.2015.07.008 26338127

[B20] Eur-Lex (2014). Regulation (EC) No 852/2004 of the European Parliament, and. (of)the Council of 29 April 2004 on the Hygiene of Foodstuffs. Available at: Available at: http://data.europa.eu/eli/reg/2004/852/oj/eng (accessed February 3, 2020) 10.1016/j.fm.2015.07.008 26338127

[B21] Fortina M. G. Ricci G. Acquati A. Zeppa G. Gandini A. Manachini P. L. (2003). Genetic characterization of some lactic acid bacteria occurring in an artisanal protected denomination origin (PDO) Italian cheese, the Toma piemontese. *Food Microbiol.* 20 397–453. 10.1016/S0740-0020(02)00149-1

[B22] Harvey J. Keenan K. P. Gilmour A. (2007). Assessing biofilm formation by *Listeria monocytogenes* strains. *Food Microbiol.* 24 380–392. 10.1016/j.fm.2006.06.006 17189764

[B23] Havelaar A. H. van Rosse F. Bucura C. Toetenel M. A. Haagsma J. A. Kurowicka D. (2010). Prioritizing emerging zoonoses in The Netherlands. *PLoS One* 5:e013965. 10.1371/journal.pone.0013965 21085625 PMC2981521

[B24] Ismaïl R. Aviat F. Michel V. Le Bayon I. Gay-Perret P. Kutnik M. (2013). Methods for recovering microorganisms from solid surfaces used in the food industry: A Review of the Literature. *Int. J. Environ. Res. Public. Health* 10 6169–6183. 10.3390/ijerph10116169 24240728 PMC3863893

[B25] Junttila J. R. Niemelä S. I. Hirn J. (1988). Minimum growth temperatures of *Listeria monocytogenes* and non-haemolytic *listeria*. *J. Appl. Bacteriol.* 65 321–327. 10.1111/j.1365-2672.1988.tb01898.x 3146567

[B26] Kusumaningrum H. D. Riboldi G. Hazeleger W. C. Beumer R. R. (2003). Survival of foodborne pathogens on stainless steel surfaces and cross-contamination to foods. *Int. J. Food Microbiol.* 85 227–236. 10.1016/S0168-1605(02)00540-8 12878381

[B27] Lagendijk E. Asséré A. Derens E. Carpentier B. (2008). Domestic refrigeration practices with emphasis on hygiene: analysis of a survey and consumer recommendations. *J. Food Prot.* 71 1898–1904. 10.4315/0362-028X-71.9.1898 18810875

[B28] Le S. Bazger W. Hill A. R. Wilcock A. (2014). Awareness and perceptions of food safety of artisan cheese makers in Southwestern Ontario: a qualitative study. *Food Control* 41 158–167. 10.1016/j.foodcont.2014.01.007

[B29] Lin C.-M. Takeuchi K. Zhang L. Dohm C. B. Meyer J. D. Hall P. A. (2006). Cross-contamination between processing equipment and deli meats by *Listeria monocytogenes*. *J. Food Prot.* 69 71–79. 10.4315/0362-028X-69.1.71 16416903

[B30] Linnan M. J. Mascola L. Lou X. D. Goulet V. May S. Salminen C. (1988). Epidemic listeriosis associated with mexican-style cheese. *N. Engl. J. Med.* 319 823–828. 10.1056/NEJM198809293191303 3137471

[B31] Lomonaco S. Gallina S. Filipello V. Leon M. S. Kastanis G. J. Allard M. (2018). Draft genome sequences of 510 *Listeria monocytogenes* strains from food isolates and human listeriosis cases from Northern Italy. *Genome Announc* 6:e01276-17 10.1128/genomeA.01276-17 PMC5773714 29348329

[B32] Mafu A. A. Plumety C. Deschênes L. Goulet J. (2011). Adhesion of pathogenic bacteria to food contact surfaces: influence of pH of culture. *Int. J. Microbiol.* 2011:972494. 10.1155/2011/972494 20981289 PMC2963129

[B33] Martinon A. Cronin U. P. Quealy J. Stapleton A. Wilkinson M. G. (2012). Swab sample preparation and viable real-time PCR methodologies for the recovery of *Escherichia coli*, *Staphylococcus aureus* or *Listeria monocytogenes* from artificially contaminated food processing surfaces. *Food Control* 24 86–94. 10.1016/j.foodcont.2011.09.007

[B34] Melo J. Andrew P. W. Faleiro M. L. (2015). *Listeria monocytogenes* in cheese and the dairy environment remains a food safety challenge: the role of stress responses. *Food Res. Int.* 67 75–90. 10.1016/j.foodres.2014.10.031

[B35] Montibus M. Ismaïl R. Michel V. Federighi M. Aviat F. Le Bayon I. (2016). Assessment of *Penicillium expansum* and *Escherichia coli* transfer from poplar crates to apples. *Food Control* 60 95–102. 10.1016/j.foodcont.2015.07.025

[B36] Morandi S. Silvetti T. Battelli G. Brasca M. (2019). Can lactic acid bacteria be an efficient tool for controlling *Listeria monocytogenes* contamination on cheese surface? The case of gorgonzola cheese. *Food Control* 96 499–507. 10.1016/j.foodcont.2018.10.012

[B37] Navas J. Ortiz S. López P. López V. Martínez-Suárez J. V. (2007). Different enrichment procedures for recovery of *Listeria monocytogenes* from raw chicken samples can affect the results of detection (by chromogenic plating or real-time PCR) and lineage or strain identification. *J. Food Prot.* 70 2851–2854. 10.4315/0362-028X-70.12.2851 18095442

[B38] Ndraha N. Hsiao H.-I. Vlajic J. Yang M.-F. Lin H.-T. V. (2018). Time-temperature abuse in the food cold chain: review of issues, challenges, and recommendations. *Food Control* 89 12–21. 10.1016/j.foodcont.2018.01.027

[B39] Patrignani F. Siroli L. Gardini F. Lanciotti R. (2016). Contribution of two different packaging material to microbial contamination of peaches: Implications in their microbiological quality. *Front. Microbiol.* 7:38 10.3389/fmicb.2016.00938 PMC4909747 27379067

[B40] Poimenidou S. V. Dalmasso M. Papadimitriou K. Fox E. M. Skandamis P. N. Jordan K. (2018). Virulence gene sequencing highlights similarities and differences in sequences in *Listeria monocytogenes* serotype 1/2a and 4b strains of clinical and food origin from 3 different geographic locations. *Front. Microbiol.* 9:1103 10.3389/fmicb.2018.01103 PMC5996115 29922249

[B41] Powell M. Schlosser W. Ebel E. (2004). Considering the complexity of microbial community dynamics in food safety risk assessment. *Int. J. Food Microbiol.* 90 171–179. 10.1016/S0168-1605(03)00106-5 14698098

[B42] Rios V. M. Dalgaard P. (2018). Prevalence of *Listeria monocytogenes* in European cheeses - A systematic review and meta-analysis. *Food Control* 84 205–214. 10.1016/j.foodcont.2017.07.020

[B43] Schirmer B. C. T. Heir E. Møretrø T. Skaar I. Langsrud S. (2013). Microbial background flora in small-scale cheese production facilities does not inhibit growth and surface attachment of *Listeria monocytogenes*. *J. Dairy Sci.* 96 6161–6171. 10.3168/jds.2012-6395 23891302

[B44] Schoder D. Stessl B. Szakmary-Brändle K. Rossmanith P. Wagner M. (2014). Population diversity of *Listeria monocytogenes* in quargel (acid curd cheese) lots recalled during the multinational listeriosis outbreak 2009/2010. *Food Microbiol.* 39 68–73. 10.1016/j.fm.2013.11.006 24387854

[B45] Siroli L. Patrignani F. Serrazanetti D. I. Chiavari C. Benevelli M. Grazia L. (2017). Survival of spoilage and pathogenic microorganisms on cardboard and plastic packaging materials. *Front. Microbiol.* 8:2606 10.3389/fmicb.2017.02606 PMC5743701 29312271

[B46] Tompkin R. B. (2002). Control of *Listeria monocytogenes* in the food-processing environment. *J. Food Prot.* 65 709–725. 10.4315/0362-028X-65.4.709 11952224

[B47] Vegara A. Rita Festino A. Di Ciccio P. Costanzo C. Pennisi L. Ianieri A. (2014). The management of the domestic refrigeration: microbiological status and temperature. *Br. Food J.* 116 1047–1057. 10.1108/BFJ-05-2012-0103

[B48] Vogel B. F. Hansen L. T. Mordhorst H. Gram L. (2010). The survival of *Listeria monocytogenes* during long term desiccation is facilitated by sodium chloride and organic material. *Int. J. Food Microbiol.* 140 192–200. 10.1016/j.ijfoodmicro.2010.03.035 20471709

[B49] Wilks S. A. Michels H. T. Keevil C. W. (2006). Survival of *Listeria monocytogenes* Scott A on metal surfaces: Implications for cross-contamination. *Int. J. Food Microbiol.* 111 93–98. 10.1016/j.ijfoodmicro.2006.04.037 16876278

[B50] Zilelidou E. A. Skandamis P. N. (2018). Growth, detection and virulence of Listeria monocytogenes in the presence of other microorganisms: microbial interactions from species to strain level. *Int. J. Food Microbiol.* 277 10–25. 10.1016/j.ijfoodmicro.2018.04.011 29677551

